# Parasitic nematodes of marine fishes from Palmyra Atoll, East Indo-Pacific, including a new species of *Spinitectus* (Nematoda, Cystidicolidae)

**DOI:** 10.3897/zookeys.892.38447

**Published:** 2019-11-27

**Authors:** David González-Solís, Lilia C. Soler-Jiménez, M. Leopoldina Aguirre-Macedo, John P. McLaughlin, Jenny C. Shaw, Anna K. James, Ryan F. Hechinger, Armand M. Kuris, Kevin D. Lafferty, Víctor M. Vidal-Martínez

**Affiliations:** 1 El Colegio de la Frontera Sur, unidad Chetumal. Av. Centenario Km 5.5, Chetumal, Quintana Roo 77014, México; 2 Institute of Parasitology, Biology Centre of the Academy of Sciences of the Czech Republic, Branišovská 31, 370 05 České Budějovice, Czech Republic; 3 Laboratorio de Patología Acuática, Centro de Investigación y de Estudios Avanzados, Mérida. Antigua carretera a Progreso km. 6, Cordemex, Mérida, Yucatán, México; 4 Department of Ecology, Evolution and Marine Biology and Marine Science Institute, University of California, Santa Barbara, CA 93106, USA; 5 Scripps Institution of Oceanography-Marine Biology Research Division, University of California, San Diego, La Jolla, California 92093, USA; 6 Western Ecological Research Center, U.S. Geological Survey, Marine Science Institute, University of California, Santa Barbara CA 93106, USA

**Keywords:** *
Cucullanus
*, *
Hysterothylacium
*, *
Paraspinitectus
*, *
Philometra
*, *
Pseudascarophis
*, *
Pulchrascaris
*

## Abstract

Here, we present the results of a taxonomic survey of the nematodes parasitizing fishes from the lagoon flats of Palmyra Atoll, Eastern Indo-Pacific. We performed quantitative parasitological surveys of 653 individual fish from each of the 44 species using the intertidal sand flats that border the atoll’s lagoon. We provide morphological descriptions, prevalence, and mean intensities of the recovered seven species of adult nematode (*Pulchrascaris chiloscyllii*, Capillariidae gen. sp., *Cucullanus bourdini*, *Cucullanus oceaniensis*, *Pseudascarophis* sp., Spinitectus (Paraspinitectus) palmyraensis**sp. nov.**, *Philometra pellucida*) and three larval stages (*Pulchrascaris* sp., *Hysterothylacium* sp., *Cucullanus* sp.). We recorded: *Pulchrascaris chiloscyllii* from *Carcharhinus melanopterus*; Capillariidae gen. sp. from *Chaetodon lunula*, *Lutjanus fulvus*, and *Ellochelon vaigiensis*; *Cucullanus bourdini* from *Arothron hispidus*; *Cucullanus oceaniensis* from *Abudefduf sordidus*; *Pseudascarophis* sp. from *Chaetodon auriga*, *Chaetodon lunula*, and *Mulloidichthys flavolineatus*; Spinitectus (Paraspinitectus) palmyraensis**sp. nov.** from *Albula glossodonta*; *Philometra pellucida* from *Arothron hispidus*; and three larval forms, *Pulchrascaris* sp. from *Acanthurus triostegus*, *Acanthurus xanthopterus*, *Rhinecanthus aculeatus*, *Platybelone argalus*, *Carangoides ferdau*, *Carangoides orthogrammus*, *Caranx ignobilis*, *Caranx melampygus*, *Caranx papuensis*, *Chaetodon auriga*, *Chanos chanos*, *Amblygobius phalaena*, *Asterropteryx semipunctata*, *Valencienea sexguttata*, *Kyphosus cinerascens*, *Lutjanus fulvus*, *Lutjanus monostigma*, *Ellochelon vaigiensis*, *Mulloidichthys flavolineatus*, *Upeneus taeniopterus*, *Gymnothorax pictus*, *Abudefduf septemfasciatus*, *Abudefduf sordidus*, and *Stegastes nigricans*; *Hysterothylacium* sp. type MD from *Acanthurus triostegus*, *Carangoides ferdau*, *Chaetodon lunula*, *Chanos chanos*, *Kyphosus cinerascens*, *Abudefduf sordidus*, and *Arothron hispidus*; and *Cucullanus* sp. from *Caranx ignobilis*. Spinitectus (Paraspinitectus) palmyraensis**sp. nov.** (Cystidicolidae) is described from the intestine of roundjaw bonefish *Albula glossodonta*. All the nematode species reported in this study represent new geographical records. We discuss how our survey findings compare to other areas of the Indo-Pacific, and the way the relatively numerical dominance of trophically transmitted larval stages likely reflect the intact food web of Palmyra Atoll, which includes a large biomass of large-bodied top predator sharks and ray-finned fishes.

## Introduction

Few studies have surveyed the parasites of all the fish species found in a habitat. In the Eastern Indo-Pacific, several studies have reported parasitic nematodes of marine fishes from Australia, French Polynesia, Okinawa (Japan), Palawan (Philippines) Indonesia, off New Caledonia,and the Hawaiian Islands (Johnston and Mawson 1951; Schmidt 1969; Deardorff et al. 1982; Lester and Sewell 1989; Bruce and Cannon 1990; Hasegawa et al. 1991; Rigby et al. 1997, 1999; Morand and Rigby 1998; Justine 2007; Lafferty et al. 2008; Moravec and Justine 2010; Palm and Bray 2014; Moravec and Justine 2018). Most studies focus on a single host species or a particular nematode genus, and a few include several large fish species (Justine 2007, 2010; Justine et al. 2012; Palm and Bray 2014). The survey by Lafferty et al. (2008) is the only one to examine parasitic nematodes of marine fishes in Palmyra Atoll, listing helminths from five fish species in the fore-reef (a habitat adjacent to the one we surveyed); however, their analysis was limited to broad patterns of richness and abundance of morphospecies, conservatively grouped into broad taxonomic categories.

Palmyra Atoll is one of the northern Line Islands located in the Eastern Indo-Pacific marine ecoregion (Spalding et al. 2007), 1680 km south-south-west of Hawaii. It is a National Wildlife Refuge managed by the US Fish and Wildlife Service (The Nature Conservancy 2006), where visitation is restricted to a small staff and a few visiting scientists or volunteers. All fishing has been prohibited at Palmyra since it became a US National Wildlife Refuge in 2000, and before that, the atoll’s remoteness kept fishing pressure low) (Stevenson et al. 2007).

This study is part of a larger project to understand the structure and function of the Palmyra Atoll’s food webs. This paper is a companion to three others examining different fish parasite taxa (Vidal-Martínez et al. 2012, 2017; Soler-Jiménez et al. 2019) from Palmyra’s lagoon flats. As such, our survey adds to the few published, detailed species descriptions or host records from this Central Indo-Pacific region (Palm and Bray 2014). The specific primary contributions of this study are 1) morphological descriptions, prevalence estimates, mean intensities, and host records for the nematode species recovered from the fish species sampled on the lagoonal flats of Palmyra Atoll, and, 2) a morphological description of a new nematode in the genus *Spinitectus*.

## Methods

Individual fish were captured between 13 October and 10 November 2009, and 22 June and 28 July 2010, by seine, spear, and hook and line from the intertidal sand flats bordering the lagoon of Palmyra Atoll (05°53'00"N, 162°05'00"W). Immediately after capture, the fish were separated and anesthetised individually with 0.5 ml/L of 2-phenoxyethanol (Sigma, St. Louis, MO, USA) in plastic bags with lagoon water and transported to the laboratory facility of the Palmyra Atoll Research Consortium (PARC). Total length and weight (g) were recorded for each individual fish. Subsequently, cavities, musculature, and all internal organs were examined for metazoan parasites in a standardized way to permit a complete estimate of the nematodes intensity (Shaw et al. 2005), using squash plates and a dissection microscope with a total magnification of 40×. Nematodes were isolated, washed in physiological saline, fixed in 4% hot formalin or 70% ethanol, labelled and stored in vials for later evaluation. The remaining specimens were flattened and cleared in a mixture of glycerine and water in different proportions, to study the morphology of structures under a compound microscope (Olympus BX-53, Olympus Corporation, Tokyo, Japan). For scanning electron microscopy (SEM), specimens were postfixed in 1% osmium tetroxide (in phosphate buffer), dehydrated through a graded acetone series, critical-point-dried, and sputter-coated with gold; they were examined using a JEOL JSM-7401F scanning electron microscope at an accelerating voltage of 4 kV (GB low mode). Measurements were made on the images at 1,000× magnification using ImageJ software (v. 1.43, April 2010). All measurements are in micrometres, unless otherwise indicated. Prevalence and mean intensity concepts were applied following Bush et al. (1997). Type and voucher specimens of each species were deposited in the Helminthological Collection of the Laboratory of Parasitology, at Centre for Research and Advanced Studies, National Polytechnic Institute, Mérida, Yucatán, México (CHCM).

## Results

A total of 653 individual fish from 44 species were examined (Table 1), 28 of which were parasitized by at least one parasitic nematode species (Table 2). *Abudefduf sordidus* (Forsskål) (Pomacentridae), *Arothron hispidus* (Linnaeus) (Tetraodontidae) and *Chaetodon lunula* (Lacepède) (Chaetodontidae) each harbored three nematode species. *Acanthurus triostegus* (Linnaeus) (Acanthuridae), *Carangoides ferdau* (Forsskål) (Carangidae), *Chaetodon auriga* Forsskål (Chetodontidae), *Chanos chanos* (Forsskål) (Chanidae), *Kyphosus cinerascens* (Forsskål) (Kiphosidae), *Ellochelon vaigiensis* (Quoy & Gaimard) (Mugilidae), *Lutjanus fulvus* (Forster) (Lutjanidae) and *Mulloidichthys flavolineatus* (Lacepède) (Mullidae) served as host for two nematode species. All other infected fish species hosted a single nematode species. Sixteen fish species were found free of any nematode parasite (Table 1).

A total of 10 nematode taxa belonging to six families were found. Seven nematode species were adults and three were larvae (Table 2). A brief taxonomic description of each species with their respective prevalences and mean intensities is presented below.

**Table 1.** Fish species examined from the lagoon flats of the Palmyra Atoll. *N* = number of fish examined; Max = maximum length reported for that fish species in FishBase (http://www.fishbase.se); range = total length range of the fish examined.

Host examined	Fish common name	*N*	Infected hosts	Max (cm)	Range (cm)
** Acanthuridae **					
*Acanthurus triostegus* (Linnaeus, 1758)	Convict surgeonfish	50	7	27	10–18
*Acanthurus xanthopterus* Valenciennes, 1835	Yellowfin surgeonfish	20	3	70	20–40
** Albulidae **					
*Albula glossodonta* (Forsskål, 1775)	Roundjaw bonefish	24	13	90	37–58
** Apogonidae **					
*Cheilodipterus quinquelineatus* Cuvier, 1828	Five-lined cardinalfish	5	0	13	5–6
** Balistidae **					
*Pseudobalistes flavimarginatus* (Rüppell, 1829)	Yellowmargin triggerfish	4	0	60	17–53
*Rhinecanthus aculeatus* (Linnaeus, 1758)	White-banded triggerfish	18	2	30	8–24
** Belonidae **					
*Platybelone argalus argalus* (Lesueur, 1821)	Keeltail needlefish	2	1	50	9–36
** Carangidae **					
*Carangoides ferdau* (Forsskål, 1775)	Blue trevally	5	4	75	33–38
*Carangoides orthogrammus* (Jordan & Gilbert, 1882)	Island trevally	3	2	75	25–35
*Caranx ignobilis* (Forsskål, 1775)	Giant trevally	4	4	170	56–79
*Caranx melampygus* Cuvier, 1833	Bluefin trevally	6	6	117	31–66
*Caranx papuensis* Alleyne & MacLeay, 1877	Brassy trevally	5	2	88	12–41
** Carcharhinidae **					
*Carcharhinus melanopterus* (Quoy & Gaimard, 1824)	Blacktip reef shark	5	4	200	46–219
** Chaetodontidae **					
*Chaetodon auriga* Forsskål, 1775	Threadfin butterflyfish	13	5	23	12–19
*Chaetodon lunula* (Lacepède, 1802)	Raccoon butterflyfish	14	11	20	11–16
** Chanidae **					
*Chanos chanos* (Forsskål, 1775)	Milkfish	5	2	180	31–57
** Gobiidae **					
*Amblygobius phalaena* (Valenciennes, 1837)	Whitebarred goby	18	6	15	1.3–7
*Asterropteryx semipunctata* Rüppell, 1830	Starry goby	12	1	6	2–4
*Gnatholepis anjerensis* (Bleeker, 1851)	Eye-bar goby	2	0	8	2–3
*Istigobius decoratus* (Herre, 1927)	Decorated goby	5	0	13	7–11
*Istigobius ornatus* (Rüppell, 1830)	Ornate goby	26	0	11	3–6
*Istigobius rigilius* (Herre, 1953)	Rigilius goby	1	0	11	4
*Oplopomus oplopomus* (Valenciennes, 1837)	Spinecheek goby	26	0	10	2–7
*Psilogobius prolatus* Watson & Lachner, 1985	Longjaw shrimpgoby	11	0	6	2–4
*Valenciennea sexguttata* (Valenciennes, 1837)	Sixspot goby	14	1	14	2–9
** Hemiramphidae **					
*Hemiramphus depauperatus* Lay & Bennett, 1839	Tropical half-beak fish	20	0	40	20–34
** Kyphosidae **					
*Kyphosus cinerascens* (Forsskål, 1775)	Blue sea chub	2	2	50	35–38
** Lutjanidae **					
*Lutjanus fulvus* (Forster, 1801)	Blacktail snapper	26	13	40	7–26
*Lutjanus monostigma* (Cuvier, 1828)	One spot snapper	6	3	60	17–37
** Mugilidae **					
*Crenimugil crenilabis* (Forsskål, 1775)	Fringelip mullet	42	0	60	8–45
*Ellochelon vaigiensis* (Quoy & Gaimard, 1825)	Squaretail mullet	54	7	63	3–32
*Osteomugil engeli* (Bleeker, 1858)	Kanda	63	0	30	1–20
** Mullidae **					
*Mulloidichthys flavolineatus* (Lacepède, 1801)	Yellowstripe goatfish	52	6	43	8–37
*Upeneus taeniopterus* Cuvier, 1829	Finstripe goatfish	5	3	33	1–30
** Muraenidae **					
*Gymnothorax pictus* (Ahl, 1789)	Paintspotted moray	7	1	140	41–70
** Ophichthidae **					
*Myrichthys colubrinus* (Boddaert, 1781)	Harlequin snake eel	3	0	97	33–65
** Pinguipedidae **					
*Parapercis lata* Randall & McCosker, 2002	Y-barred sandperch	13	0	21	2–3
** Pomacentridae **					
*Abudefduf septemfasciatus* (Cuvier, 1830)	Banded sergeant	12	1	23	14–20
*Abudefduf sordidus* (Forsskål, 1775)	Blackspot sergeant	18	4	24	14–19
*Chrysiptera glauca* (Cuvier, 1830)	Grey demoiselle	3	0	12	8–10
*Stegastes nigricans* (Lacepède, 1802)	Dusky farmerfish	10	1	14	8–10
** Serranidae **					
*Epinephelus merra* Bloch, 1793	Honeycomb grouper	2	0	32	13–24
** Sphyraenidae **					
*Sphyraena barracuda* (Edwards, 1771)	Great barracuda	2	0	200	65–76
** Tetraodontidae **					
*Arothron hispidus* (Linnaeus, 1758)	White–spotted puffer	15	7	50	17–49

**Table 2.** Parasitic nematodes of fishes from the lagoon flats of Palmyra Atoll and their infection parameters; *N* = number of fish examined. *Larval forms.

	Hosts	*N*	Infected hosts	Prevalence (%)	Mean intensity (±SD)
** Anisakidae **					
*Pulchrascaris chiloscyllii*	*Carcharhinus melanopterus*	5	4	80	35 ± 26.5
*Pulchrascaris* sp.*	*Acanthurus triostegus*	50	4	8	2.3 ± 1.9
	*Acanthurus xanthopterus*	20	3	15	1.3 ± 0.6
	*Rhinecanthus aculeatus*	18	2	11.1	1
	*Platybelone argalus*	2	1	50	10
	*Carangoides ferdau*	5	2	40	42 ± 58
	*Carangoides orthogrammus*	3	2	67	52.5 ± 72.8
	*Caranx ignobilis*	4	4	100	71.3 ± 106.5
	*Caranx melampygus*	6	6	100	53.7 ± 106.4
	*Caranx papuensis*	5	2	40	8 ± 9.9
	*Chaetodon auriga*	13	1	8	1
	*Chanos chanos*	5	1	20	2
	*Amblygobius phalaena*	18	6	33	7.2 ± 8.6
	*Asterropteryx semipunctata*	12	1	8	3
	*Valencienea sexguttata*	14	1	7	1
	*Kyphosus cinerascens*	2	1	50	7
	*Lutjanus fulvus*	26	12	46	5.2 ± 3.7
	*Lutjanus monostigma*	6	3	50	4.7 ± 3.2
	*Ellochelon vaigiensis*	54	4	7	1.5 ± 0.6
	*Mulloidichthys flavolineatus*	52	5	10	1.4 ± 0.4
	*Upeneus taeniopterus*	5	3	60	3.7 ± 1.5
	*Gymnothorax pictus*	7	1	14	3
	*Abudefduf septemfasciatus*	12	1	8	1
	*Abudefduf sordidus*	18	1	6	1
	*Stegastes nigricans*	10	1	10	1
*Hysterothylacium* sp. type MD^*^	*Acanthurus triostegus*	50	3	6	1.7 ± 0.6
	*Carangoides ferdau*	5	2	40	1.0
	*Chaetodon lunula*	14	7	50	3.3 ± 1.6
	*Chanos chanos*	5	1	20	6
	*Kyphosus cinerascens*	2	1	50	2
	*Abudefduf sordidus*	18	1	6	3
	*Arothron hispidus*	15	3	20	7.0 ± 5.0
** Capillariidae **					
Capillariidae gen. sp.	*Chaetodon lunula*	14	2	14	2.5 ± 0.7
	*Lutjanus fulvus*	26	1	4	1
	*Ellochelon vaigiensis*	54	3	6	1.7 ± 0.6
** Cucullanidae **					
*Cucullanus bourdini*	*Arothron hispidus*	15	7	47	6 ± 4.7
*Cucullanus oceaniensis*	*Abudefduf sordidus*	18	2	11	1.5 ± 0.7
*Cucullanus* sp.^*^	*Caranx ignobilis*	4	1	25	2
** Cystidicolidae **					
Spinitectus (Paraspinitectus) palmyraensis sp. nov.	*Albula glossodonta*	24	13	54	3.2 ± 4.0
*Pseudascarophis* sp.	*Chaetodon auriga*	13	4	31	1
	*Chaetodon lunula*	14	2	14	3 ± 2.8
	*Mulloidichthys flavolineatus*	52	1	2	1
** Philometridae **					
*Philometra pellucida*	*Arothron hispidus*	15	6	40	20.8 ± 31.4

### Family Anisakidae Railliet & Henry, 1912

#### *Pulchrascaris* Vicente & Santos, 1972

##### Pulchrascaris chiloscyllii

Taxon classificationAnimaliaSpiruridaCystidicolidae

(Johnston & Mawson, 1951) Deardorff, 1987

B789CA60-3CD3-5D0B-8D62-C6DF2C899527

###### Description.

Male (1 mature specimen, measurements of one young specimen in parenthesis): elongate, relatively large nematodes, 68.01 (20.36) mm long, 767 (388) wide. Cuticular alae distinct. Lips small, greatly reduced, with tooth-like structures. Nerve ring located at first third of esophagus length, 503 (380); deirids posterior to level of nerve ring, 604, both from anterior body end. Excretory pore between base of subventral lips. Esophagus cylindrical, 2.96 (1.95) mm long, with large ventriculus at posterior end, 3.38 (2.12) mm long, and without ventricular appendage. Intestinal caecum anteriorly directed, somewhat larger than ventriculus, 3.57 (2.36) mm long. Spicules equal, similar, alate, 1.48 mm long; gubernaculum absent. Caudal papillae 51 pairs: 42 precloals pairs (some subventral and other sublateral), 4 adcloacal subventral pairs, 5 postcloacal (including phasmids). One single, ventral papilla on the anterior cloacal lip. Three cuticular plates immediately posterior to cloacal opening, with serrate edges. Tail conical, short, 298 (182) long.

Fourth-stage larva (1 specimen): Length of body 11.56 mm long, 233 wide. Esophagus, ventriculus and intestinal caecum 1.25, 0.93, 1.08 mm, respectively. Nerve ring 275, deirids 355, both from the cephalic end. Tail 126 long.

###### Host.

*Carcharhinus melanopterus* (Quoy & Gaimard) (Carcharhinidae).

###### Site of infection.

Intestine.

###### Prevalence and mean intensity.

80 and 35 ± 26.5 (*n* = 5).

###### Specimens deposited.

CHCM no. 619 (voucher) (1 vial, 1 specimen ♀).

###### Remarks.

These specimens are morphologically similar to *P. chiloscyllii* (reported as *Terranova chiloscyllii*), originally found in *Chiloscyllium punctatum* Müller & Henle and *Mustelus antarcticus* Günther (reported as *Emissola antarctica*) from the Central Queensland coast (Johnston and Mawson 1951). This species was redescribed by Deardorff (1987) from *Sphyrna lewini* (Griffith) and *Sphyrna zygaena* (Linnaeus) (Sphyrnidae) off Hawaii, Alabama and South Africa, and later recorded in *Trianenodon obesus* (Rüppell) (Carcharhinidae) and *C. melanopterus* from the Solomon Islands and the Maldives by Bruce and Cannon (1990). Our specimens are somewhat larger than those described earlier, but this difference is consistent with intraspecific variability.

##### Pulchrascaris

Taxon classificationAnimaliaSpiruridaCystidicolidae

sp.

2222A03C-4943-596F-A93E-31D27BEFB94B

###### Description.

Third-stage larva (12 specimens): relatively long nematodes 6.99–12.42 mm long and 172–452 wide, whitish with lateral cuticular alae. Lips weakly developed and bearing a larval, ventral tooth, 5–12 long. Nerve ring encircling esophagus in its anterior third, 173–302 from anterior body end. Deirids slightly posterior to nerve ring, 225–273 from cephalic end. Excretory pore at base of subventral lip. Esophagus relatively long, 636–1,414 with elongated ventriculus at posterior end, 388–1,371 long. Intestinal caecum anteriorly directed and somewhat larger than ventriculus, 389–1,384 long. Tail conical, 99–179 long, without ornamentations. Some specimens with a single papilla at 1.65–5.78 mm from posterior end of body.

###### Hosts.

*Acanthurus sordidus*, *A. triostegus*, *Acanthurus xanthopterus* Valenciennes (Acanthuridae), *Rhinecanthus aculeatus* (Linnaeus) (Balistidae), *Platybelone argalus argalus* (Lesueur) (Belonidae), *C. ferdau*, *Carangoides orthogrammus* (Jordan & Gilbert), *Caranx ignobilis* (Forsskål), *Caranx melampygus* Cuvier, *Caranx papuensis* Alleyne & MacLeay (all Carangidae), *Amblygobius phalaena* (Valenciennes), *Asterropteryx semipunctata* Rüppell, *Valenciennea sexguttata* (Valenciennes) (all Gobiidae), *M. flavolineatus*, *Upeneus taeniopterus* Cuvier (Mullidae), *Gymnothorax pictus* (Ahl) (Muraenidae), *L. fulvus*, *Lutjanus monostigma* (Cuvier) (Lutjanidae), *Abudefduf septemfasciatus* (Cuvier), *Stegastes nigricans* (Lacepède) (Pomacentridae), *C. auriga*, *C. chanos*, *K. cinerascens*, *E. vaigiensis*.

###### Site of infection.

Mesenteries.

###### Prevalence and mean intensity.

5.6 and 1 (*n* = 18) to *A. sordidus*, 8 and 2.3 ± 1.9 (*n* = 50) to *A. triostegus*, 15 and 1.3 ± 0.6 (*n* = 20) to *A. xanthopterus*, 11.1 and 1 (*n* = 18) to *R. aculeatus*, 50 and 10 (*n* = 2) to *P. argalus*, 40 and 42 ± 58 (*n* = 5) to *C. ferdau*, 66.7 and 52.5 ± 72.8 (*n* = 2) to *C. orthogrammus*, 100 and 71.3 ± 106.5 (*n* = 4) to *C. ignobilis*, 100 and 53.7 ± 106.4 (*n* = 6) to *C. melampygus*, 40 and 8 ± 9.9 (*n* = 5) to *C. papuensis*, 33.3 and 7.2 ± 8.6 (*n* = 18) to *A. phalaena*, 8.3 and 3 (*n* = 12) to *A. semipunctata*, 7.1 and 1 (*n* = 14) to *V. sexguttata*, 9.6 and 1.4 ± 0.4 (*n* = 52) to *M. flavolineatus*, 60 and 3.7 ± 1.5 (*n* = 5) to *U. taeniopterus*, 14.3 and 3 (*n* = 7) to *G. pictus*, 46.2 and 5.2 ± 3.7 (*n* = 26) to *L. fulvus*, 50 and 4.7 ± 3.2 (*n* = 6) to *L. monostigma*, 8.3 and 1 (*n* = 12) to *A. septemfasciatus*, 10 and 1 (*n* = 10) to *S. nigricans*, 7.7 and 1 (*n* = 13) to *C. auriga*, 20 and 2 (*n* = 5) to *C. chanos*, 50 and 7 (*n* = 2) to *K. cinerascens*, 7.4 and 1.5 ± 0.6 (*n* = 54) to *E. vaigiensis*.

###### Specimens deposited.

CHCM no. 620 (voucher) (1 vial, 1 specimen ♀) (from *A. triostegus*), CHCM no. 621 (voucher) (1 vial, 2 specimens ♂ ♀) (from *C. auriga*), CHCM no. 622 (voucher) (1 vial, 1 specimen ♀) (from *K. cinerascens*), CHCM no. 623 (voucher) (1 vial, 1 specimen ♀) (from *L. fulvus*).

###### Remarks.

These larvae belong to the genus *Pulchrascaris* because of the position of the excretory pore between the subventral lips, absence of ventricular appendage, elongate esophagus, relatively large ventriculus, and intestinal caecum anteriorly directed and somewhat larger than ventriculus. Deardorff (1987) mentioned that differentiation of third stage larvae of the genera *Pulchrascaris* and *Terranova* is difficult, since lips are not well developed at that stage. We found one adult male, a young female and one larva of the species *P. chiloscyllii* in the blacktip reef shark *C. melanopterus*, which occurs in the same area and feeds on some small reef fishes (Froese and Pauly 2014). Larvae identified as *Pulchrascaris* sp. are identical to those in the shark, and they could belong to the same species, but until studies on the life cycle or molecular analysis are carried out, we considered them as separate taxa. All fishes reported here probably act as intermediate or paratenic hosts and elasmobranchs are the definitive hosts. Jabbar et al. (2012) reported larval anisakid nematodes from several teleosts from the Great Barrier Reef, including larvae named as *Terranova* sp. type II in *C. papuensis*. Those nematodes differ from the present ones in the length and ratio of the intestinal caecum and ventriculus. Larval nematodes reported as *Terranova* sp. type I and *Terranova* sp. type Hawaii B (HB) (Cannon 1977; Deardorff et al. 1982; Palm and Bray 2014) should be considered as *Pulchrascaris* sp. according to the ratios of their esophageal appendages. All fish parasitized by these larvae in the present work represent new host records.

### Family Raphidascarididae Hartwich, 1954

#### Hysterothylacium

Taxon classificationAnimaliaSpiruridaCystidicolidae

sp. type MD of Deardorff & Overstreet, 1981 (larval type VIII of Shamsi et al., 2011)

95D0CB73-8270-53CC-B339-FE5DC8D611AF

##### Description.

Third-stage larva (5 specimens): medium-sized nematodes, 3.00–8.64 mm long, 100–267 wide. Cuticular lateral alae extending along whole length of worm. Poorly developed lips, small, 18 long and 19 wide. Esophagus 270–680 long, with almost spherical ventriculus at distal part, 59–75 long and 57–87 wide. Ventricular appendage 481–595 long; intestinal caecum small, anteriorly directed, 67–274 long. Ratio for length of ventricular appendage and intestinal caecum 1: 2.10–7.73. Nerve ring at 116–279 from anterior end of body. Excretory pore slightly anterior to nerve ring, 137–198 from cephalic end. Deirids 395 from anterior end (observed in only 1 specimen). Tail conical, 121–227 long, with small spine-like mucron at tip.

##### Hosts.

*Acanthurus triostegus*, *C. ferdau*, *C. lunula*, *C. chanos*, *K. cinerascens*, *A. sordidus*, and *A. hispidus*.

##### Site of infection.

Mesenteries and liver.

##### Prevalence and mean intensity.

6 and 1.7 ± 0.6 (*n* = 50) to *A. triostegus*, 40 and 1.0 ± 0.0 (*n* = 5) to *C. ferdau*, 50 and 3.3 ± 1.6 (*n* = 14) to *C. lunula*, 20 and 6 (*n* = 5) to *C. chanos* and 50 and 2 (*n* = 2) to *K. cinerascens*, 5.6 and 3 (*n* = 18) to *A. sordidus*, 20 and 7.0 ± 5.0 (*n* = 15) to *A. hispidus*.

##### Specimens deposited.

CHCM no. 624 (voucher) (1 vial, 1 specimen ♀) (from *Chaetodon lunula*), CHCM no. 625 (voucher) (1 vial, 2 specimens ♂ ♀) (from *K. cinerascens*).

##### Remarks.

Because of the presence of a small intestinal caecum, long ventricular appendage, rounded ventriculus and small mucron on the tail tip, these larvae are morphologically similar to those described by Deardorff and Overstreet (1981) and the type HA of Deardorff et al. (1982) in the Gulf of Mexico and the Hawaiian Island, respectively. Recently, Shamsi et al. (2011) proposed a new classification for larvae occuring in fishes off Australia according to their molecular characterization. Morphometrically, larvae from America and the Australian region were practically identical. *Arothron hispidus*, *C. lunula*, and *K. cinerascens* represent new host records.

### Family Capillariidae Railliet, 1915

#### Capillariidae

Taxon classificationAnimaliaSpiruridaCystidicolidae

gen. sp.

5DDBE2B3-695F-5E90-994F-181A167FCD3E

##### Description.

Gravid female (1 damaged specimen, measurements of 1 young female in parentheses): long, thin and slender nematodes, 9.42 (8.80) mm long and 26 (37) wide. Muscular esophagus – (335). Stichosome formed by 4–6 (5) stichocytes. Eggs with polar plugs, thick-walled, 49–55 × 20–24 (–). Tail very short, 7 (6) long.

##### Hosts.

*Chaetodon lunula*, *L. fulvus*, and *E. vaigiensis*.

##### Site of infection.

Stomach.

##### Prevalence and mean intensity.

14.3 and 2.5 ± 0.7 (*n* = 14) to *C. lunula*, 3.8 and 1 (*n* = 26) to *L. fulvus*, 5.6 and 1.7 ± 0.6 (*n* = 54) to *E. vaigiensis*.

##### Specimens deposited.

CHCM no. 626 (voucher) (1 vial, 1 specimen ♀) (from *Chaetodon lunula*), CHCM no. 627 (voucher) (1 vial, 2 specimens ♂ ♀) (from *L. fulvus*).

##### Remarks.

Specimens were damaged, but it was possible to observe the most important features to allocate them to the family Capillariidae, such as esophagus divided in muscular and glandular parts (stichosome), eggs with polar plugs, and the general shape of body. Since males are unknown, it is impossible to determine their generic or specific identity. This is the first capillariid nematode reported in *C. ignobilis* and the second for the family Carangidae in the southwestern Pacific Ocean, which was recorded in *Carangoides oblongus* (Cuvier) (Carangidae) off New Caledonia (Moravec and Justine 2010). Palm and Bray (2014) only recorded *Capillaria* eggs in the musculature from *Bathygobius fuscus* (Rüppell) (Gobiidae) in Hawaii.

### Family Cucullanidae Cobbold, 1864

#### Cucullanus bourdini

Taxon classificationAnimaliaSpiruridaCystidicolidae

Petter & Le Bel, 1992

C87CBBD9-F79E-52F7-B465-A7C38652B9C2

##### Description.

Male (5 specimens): medium-sized nematodes, 8.11–12.87 mm long, 223–413 wide. Esophagus 777–1,003 long and 126–240 wide at its posterior end. Anterior part of esophagus forming pseudocapsule (esophastome) 228–275 long and 129–185 wide. Nerve ring surrounding esophagus at its first third, 267–368 from anterior body end. Excretory pore beyond posterior end of esophagus, cervical papillae on distal end of esophagus or slightly posterior to it, 969–1,215 and 671–937, respectively, from cephalic end. Well-developed precloacal pseudosucker present, its distal part at 626–942 from posterior body end. Cloacal opening elevated. Eleven pairs of caudal papillae: 3 subventral, pedunculate precloacal pairs (first pair sometimes anterior to sucker and sometimes over anterior border), 4 adcloacal pairs (3 subventral, 1 lateral), 4 postcloacal pairs, including phasmids (1 subdorsal, 1 lateral, 2 subventral). One single papilla on anterior cloacal lip. Spicules similar, equal, alate, 982–1,543 with supporting structures along almost their whole length. Gubernaculum well-sclerotized, 116–140 long. Right and left postdeirids 3.94–6.37 and 1.92–3.27 mm, respectively, from posterior end of body. Tail conical 187–236 long, with pointed tip.

Gravid females (5 specimens, measurements of 1 young female in parentheses): body length 13.72–15.90 (5.23) mm long and 472–500 (220) wide. Esophagus 999–1,087 (600) long, 227–263 (106) wide at its posterior end. Esophastome 250–311 (197) long, 169–201 (129) wide. Nerve ring, excretory pore and deirids 318–367 (250), 1,150–1,377 (902), 805–971 (614), respectively, from anterior end of body. Vulva postequatorial 7.97–9.35 (3.30) from anterior end of body, with somewhat elevated lips and posteriorly directed muscular vagina. Eggs oval, 53–71 × 34–46 (–) in size, non-larvated. Tail conical, 294–385 (204) long.

##### Host.

*Arothron hispidus*.

##### Site of infection.

Intestine.

##### Prevalence and mean intensity.

46.7 and 6 ± 4.7 (*n* = 15).

##### Specimens deposited.

CHCM no. 628 (voucher) (1 vial, 1 specimen ♀).

##### Remarks.

The measurements of these nematodes are similar to those of *C. bourdini*, a species described from lutjanid fishes off New Caledonia (Petter and Le Bel 1992; Moravec and Justine 2011), although it was also reported from *Balistapus undulatus* Park (Balistidae) and *Myripristis kuntee* Valenciennes (Holocentridae) from the French Polynesia (Morand and Rigby 1998). They have overlapping measurements of the body length of males, spicules, gubernaculum, and similar number and distribution of caudal papillae, although there are some differences in female body length, which could be considered to represent intraspecific variability. Apparently, this nematode is not host-specific (see Moravec and Justine 2011), since it has been reported from four different fish families to date. This is the first record of *C. bourdini* in *A. hispidus* and the second for a tetraodontiform fish.

#### Cucullanus oceaniensis

Taxon classificationAnimaliaSpiruridaCystidicolidae

Moravec, Sasal, Würtz & Taraschewski, 2005

93F46033-05BD-5F4A-9956-CA2D277F01D0

##### Description.

Male (1 specimen): medium-sized nematodes, whitish. Body length 6.22 mm, 183 wide. Muscular esophagus 678 long and 112 wide at its posterior part. Anterior end of esophagus forming a pseudobuccal capsule (esophastome), 214 long and 153 wide. Nerve ring slightly posterior to esophastome, 275 from anterior end of body. Deirids anterior to posterior end of esophagus, excretory pore posterior to it, at 609 and 797, respectively, from cephalic end. Eleven pairs of caudal papillae (including phasmids): 3 subventral precloacal pairs, 4 adcloacal pairs (3 subventral, 1 lateral), 4 postcloacal pairs (3 subventral, 1 lateral). A single, ventral papilla on the anterior cloacal lip. Spicules equal, similar, alate, 374 long. Gubernaculum well sclerotized, 112 long. Ventral precloacal sucker well developed, posterior margin at 690 from posterior end of body. Postdeirids 1.71 mm from tail tip. Tail conical with pointed tip, 112 long.

##### Host.

*Abudefduf sordidus*.

##### Site of infection.

Intestine.

##### Prevalence and mean intensity.

11.1 and 1.5 ± 0.7 (*n* = 18).

##### Specimens deposited.

CHCM no. 629 (voucher) (1 vial, 1 specimen ♀).

##### Remarks.

This single male is morphologically similar to *C. bourdini* and *C. oceaniensis*, two species described from lutjanids off New Caledonia and *Anguilla marmorata* Quoy & Gaimard (Anguillidae) in Polynesia and Melanesia (Petter and Le Bel 1992; Moravec et al. 2005). The number and distribution of caudal papillae of this male are practically the same as those of the above-mentioned species, although much more similar to that of *C. oceaniensis*. However, we found some differences in body (6.22 vs 10.6–14.0 and 7.14–9.51 mm) and spicule lengths (374 vs 740–1,000 and 819–1,020 mm) of the present male. These differences could be related to the suitability of the fish hosts, since apparently the adult males in *C. bourdini* and *C. oceaniensis* are able to fully develop in lutjanids, while *A. sordidus* might act as a paratenic or not preferential host for this nematode, since maturity was not fully reached. According to the original descriptions of *C. bourdini* and *C. oceaniensis*, these two species share many morphometric values and have never been compared (see Petter and Le Bel 1992; Moravec et al. 2005; Moravec and Justine 2011). An examination of the type material of both species should be carried out to elucidate their possible synonymy.

#### Cucullanus

Taxon classificationAnimaliaSpiruridaCystidicolidae

sp.

B65BE845-6257-5FEB-925F-17222307EE95

##### Description.

Third-stage larva (2 specimens): small nematodes, 1.45–1.53 long, 50– 51 wide. Esophagus 333–352 long, 38–39 wide at its posterior part. Esophastome 60–65 long and 44–50 wide. Nerve ring 139–151, excretory pore 374–413 and deirids 262. Excretory pore posterior to distal end of esophagus, deirids on its posterior end of esophagus or slightly anterior to it. Tail elongate, 105–113 long, with pointed tip.

##### Host.

*Caranx ignobilis*

##### Site of infection.

Intestine.

##### Prevalence and mean intensity.

25 and 2 (*n* = 4).

##### Specimens deposited.

CHCM no. 630 (voucher) (1 vial, 1 specimen ♀).

##### Remarks.

These were two very small and poorly developed cucullanid larvae and it was not possible to identify them to species. Perhaps, they represent new infections, or this fish acts as paratenic host. These are the first cucullanid nematodes reported from *C. ignobilis* and the family Carangidae in the Indo-Pacific Ocean.

### Family Cystidicolidae Skryabin, 1946

#### Pseudascarophis

Taxon classificationAnimaliaSpiruridaCystidicolidae

sp.

75B8D7F5-3992-5DB7-8499-99404A8475BB

##### Description.

Gravid female (1 specimen): large, whitish nematode, 18.20 mm long, 158 wide. Anterior end rounded with two large rounded pseudolabia. Vestibule relatively short, 159 long, with anterior prostom and posterior part forming a transverse ring on anterior end of esophagus. Muscular esophagus short, narrow, 258 long; glandular part broader, 5 times longer than muscular one, 1.52 mm long. Nerve ring encircling muscular esophagus at its first third, 206 from anterior body end. Deirids small, bifurcated, situated between second and third thirds of vestibule length, 109 from anterior end of body. Excretory pore posterior to level of nerve ring, 220 from cephalic end. Vagina muscular directed posteriorly. Vulva pre-equatorial, 8.74 mm from anterior end of body, with not elevated lips. Fully developed eggs, thick-walled, larvated, without filaments, 31–36 × 24–29. Tail elongate, 239 long, with rounded tip.

##### Hosts.

*Chaetodon auriga*, *C. lunula*, and *M. flavolineatus*.

##### Site of infection.

Stomach.

##### Prevalence and mean intensity.

30.8 and 1 (*n* = 13) to *C. auriga*, 14.3 and 3 ± 2.8 (*n* = 14) to *C. lunula*, 1.9 and 1 (*n* = 52) *M. flavolineatus*.

##### Specimens deposited.

CHCM no. 631 (voucher) (1 vial, 1 specimen ♀) (from *C. auriga*), CHCM no. 632 (voucher) (1 vial, 2 specimens ♂ ♀) (from *M. flavolineatus*).

##### Remarks.

The presence of rounded pseudolabia, bifurcated deirids, and elongate tail with rounded tip, make this female similar to those of the genus *Pseudoascarophis*. Nematodes belonging to this genus were originally reported in *K. cinerascens* from off Japan and later found in *Parupeneus chrysopleuron* (Temminck & Schlegel) (Mullidae), *Genypterus chilensis* (Guichenot) (Ophidiidae), *Kyphosus sectatrix* (Linnaeus) (Kyphosidae) from China, Chile, Brazil, respectively (Ko et al. 1985; Solov’eva 1996; Muñoz and George-Nascimento 2001; Pereira et al. 2013). Therefore, this find represents new host and geographical records. Since only one female was recovered, it is impossible to identify it to species.

#### Spinitectus (Paraspinitectus) palmyraensis

Taxon classificationAnimaliaSpiruridaCystidicolidae

González-Solís & Vidal-Martínez sp. nov.

E645F51A-8D4B-58ED-929E-13CB1D81553E

http://zoobank.org/AB52DF93-B3F2-4C78-A221-7720368266C8

Figs 1 , 2 , 3

##### Description.

**Figure 1.**
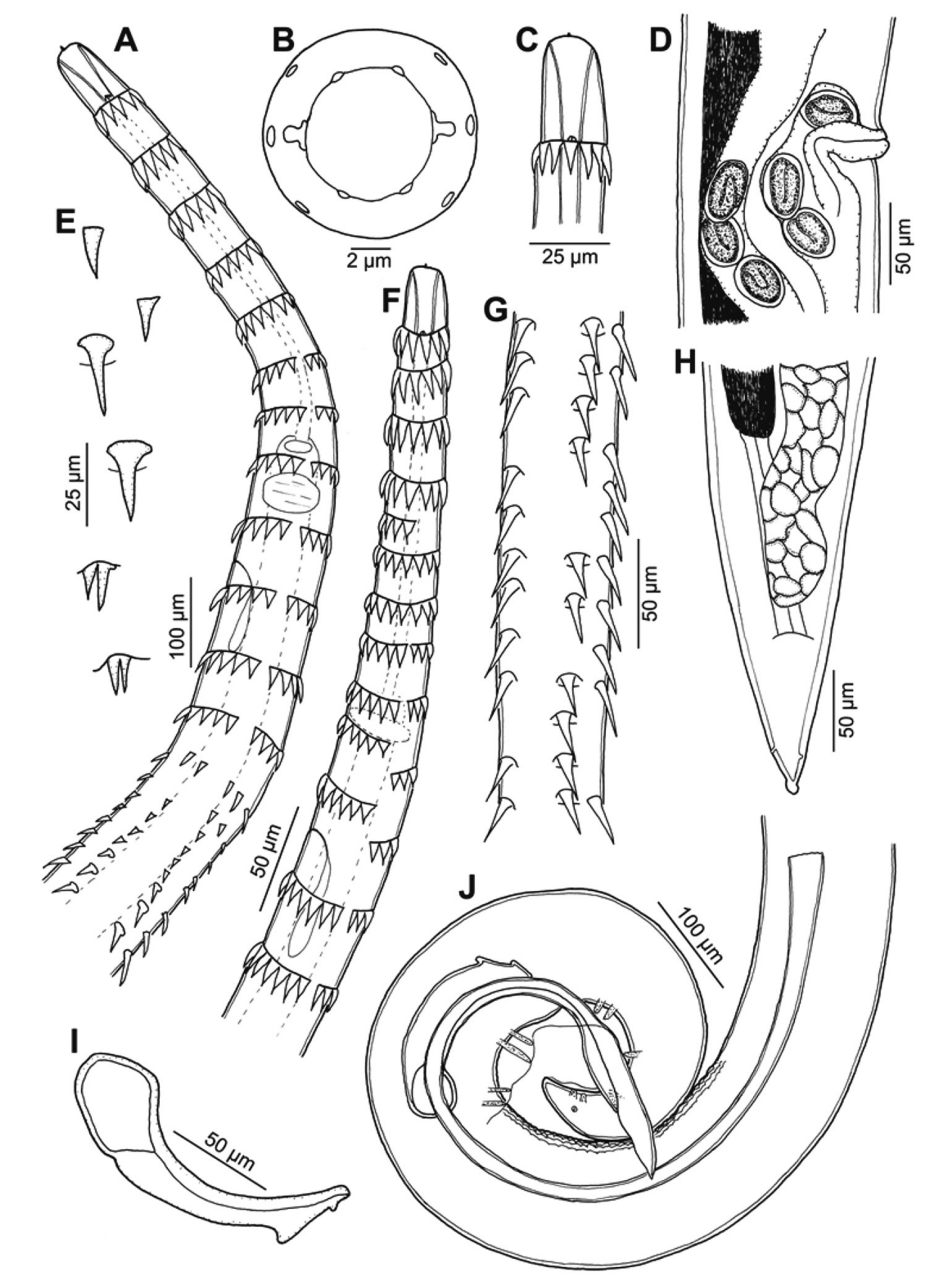
Spinitectus (Paraspinitectus) palmyraensis sp. nov. **A** anterior extremity of male, lateral view **B, C** cephalic end, apical and lateral views, respectively **D** region of vulva, lateral view **E** spines from different parts of body **F** anterior end, showing incomplete rows of spines **G** region of mid-body, showing missing spines **H** tail of female, ventral view **I** small spicule, lateral view **J** posterior end of male, lateral view.

**Figure 2.**
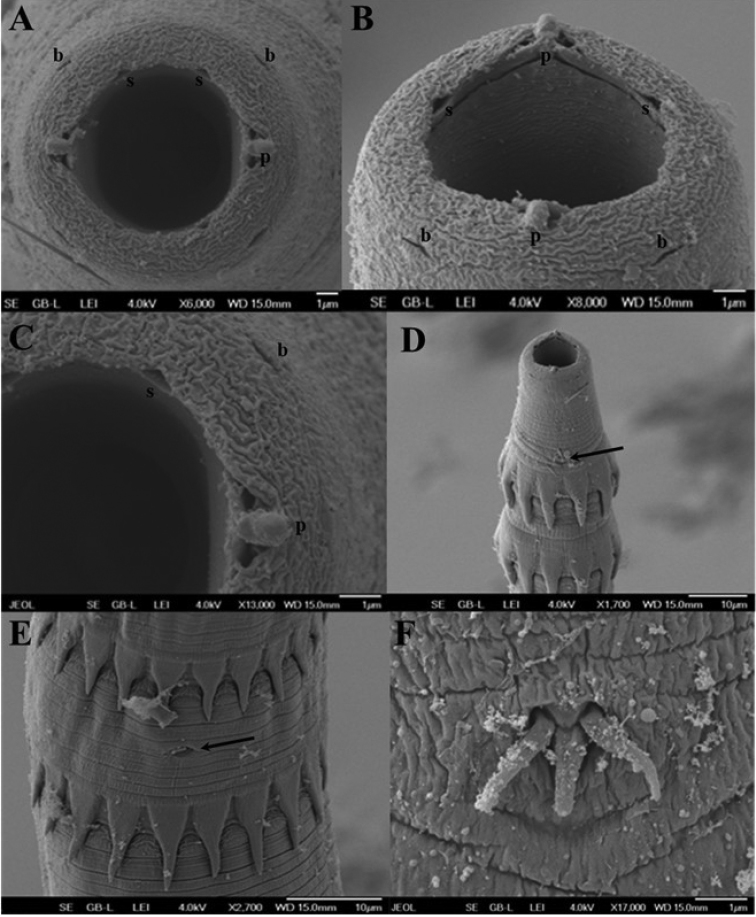
Spinitectus (Paraspinitectus) palmyraensis sp. nov. scanning electron micrographs. **A, B** anterior end of gravid female, apical and subapical views, respectively **C** detail of mouth, apical view **D** anterior end body, lateral view (arrow indicates deirids) **E** region of excretory pore, ventral view (arrow indicates the excretory pore) **F** deirids. Abbreviations: **b** submedian papilla, **l** labium, **p** pseudolabium, **s** sublabium.

**Figure 3.**
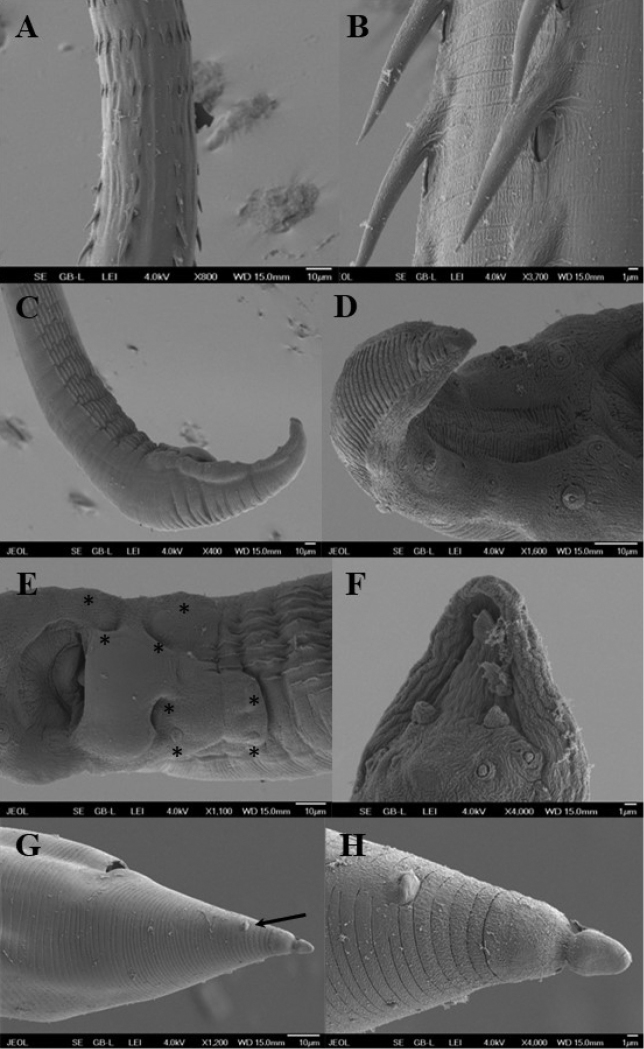
Spinitectus (Paraspinitectus) palmyraensis sp. nov. scanning electron micrographs. **A** transition zone of spination, lateral view **B** larger spines with pore-like on bases **C** posterior end of male showing area rugosa, sublateral view **D** tail of male, ventral view **E** region of cloaca, ventral view (asterisks indicate precloacal papillae) **F** tail tip of male, ventral view **G** posterior end female, lateral view (arrow indicates phasmid) **H** detail of tail tip.

***General***: medium-sized nematodes with transverse rings of markedly long, posteriorly directed spines. First ring situated at level of base of prostom (Figs 1C, 2D), other rings extending posteriorly to mid-length of body. Spination is weakly visible in the posterior end of body (Figs 1H, 3C, G). Rings 1–4 with 11–14 uninterrupted spines, rings 5–12 with 13–17 interrupted spines at lateral side of body, rings 13–14 with 13–15 discontinuous spines in number and shape (Fig. 1A), rings 15 and posteriormost rings with 6 relatively large spines with a pore-like in their bases (Fig. 3B). In some specimens, anteriormost rings incomplete, assymetrical and not forming a circle or with some missing spines (Fig. 1F, G), sometimes with double spines (Fig. 1E). Spines from rings 1–15 not overlapping each other, spines of more posterior rings overlapping (Fig. 3A). Cuticle transversely striated forming elevated rings (Figs 2D–E, 3A). The oral aperture oval surrounded by four submedian labia, which form continuous dorsal and ventral margins around the mouth. Two dorsal and two ventral submedian sublabia, curved and attached by their bases to surface of labia. There are two lateral, highly reduced pseudolabia without internal extensions. Two pairs of submedian cephalic papillae are present and a pair of lateral, barely visible amphids are situated outside the oral aperture (Figs 1B, 2A–C). Vestibule straight, rather long, with anterior end distinctly distended to form funnel-shaped prostom in lateral view (Fig. 1A, F). Esophagus clearly divided into muscular portion and posterior glandular, much longer and slightly wider portion. Nerve ring encircles muscular esophagus near its anterior end, situated between 8^th^ and 9^th^ rings of cuticular spines (Fig. 1A, F). Excretory pore situated between 9^th^ and 10^th^ rings of spines (Figs 1A, F, 2E). Small, trifurcated deirids situated just anterior to first ring of spines (Figs 1A, C, F, 2F). Tail of both sexes conical.

**Male** (4 specimens, measurements of holotype in parenthesis): length of body 3.90–5.50 (4.92) mm, maximum width 89–115 (103). First cuticular ring 31–33 (33) from anterior extremity, armed with 11–14 (12) spines, 10–14 (12) long. Larger spines 22–23 (22–23) are at the level of the glandular esophagus. Vestibule including prostom 188–213 (188) long; prostom 21–28 (28) long and 7–9 (8) wide. Muscular esophagus 251–278 (261) long; glandular esophagus 0.82–1.55 (0.93) mm long, 32–41 (33) wide; length ratio of both parts of esophagus 1:3.2–4.2 (3.5). Entire esophagus and vestibule represent 28–31 (29)% of whole body length. Nerve ring and excretory pore 212–223 (223) and 233–254 (254), respectively, from anterior extremity. Deirids 30–32 (32) from anterior end of body. Posterior end of body ventrally curved, provided with well-developed caudal alae reaching posteriorly to end of tail (Figs 1J, 3C–F). Well-developed longitudinal, ventral cuticular ridges (area rugosa) present in precloacal region, formed by 9 lines in its most anterior part, 10 at middle and 3 near cloacal opening (Figs 1J, 3C, E). Precloacal papillae: 4 pairs of subventral, pedunculate, close to each other and equally distributed papillae present. Postcloacal papillae: 6 pairs of subventral pedunculate papillae, although second pair slightly laterally displaced. One pair of lateral papillae situated between two last pairs of subventrals (probably representing phasmids) (Figs 1J, 3D–F). Spicules dissimilar and unequal; large (left) spicule 673–766 (713) long, striated, with expanded proximal end 31–39 (39) wide, and bifurcated distal part. Small (right) spicule boat-shaped, 126–155 (126) long, with narrowed, ventrally bent distal end and two dorsal protuberances (Fig. 1I, J). Length ratio of spicules 1: 4.4–5.6 (5.6). Tail 123–160 (141) long, with blunt tip (Fig. 3F).

**Gravid female** (4 specimens; measurements of allotype in parentheses): body length 5.89–7.14 (6.26) mm, maximum width 129–143 (143). First cuticular ring 27–31 (27) from anterior extremity, 11–14 spines 12–15 (12) long. Larger spines 12–14 (12–14) at the level of the glandular esophagus. Vestibule including prostom 183–211 (183) long; prostom 23–28 (28) long and 7–9 (7) wide. Muscular esophagus 273–300 (289) long; glandular esophagus 0.908–1.165 (1.165) mm long, 38–46 (46) wide; length ratio of both parts of esophagus 1: 3.3–4.1 (4). Entire esophagus and vestibule with prostome represents 23–26 (26)% of whole body length. Nerve ring and excretory pore 206–231 (206) and 219–266 (206), respectively, from anterior extremity. Deirids 26–30 (26) from anterior end. Vulva with slightly elevated lips, postequatorial, situated at 4.24–4.82 (4.58) mm from anterior extremity, representing 67–73 (73)% of body length. Vagina muscular, directed posteriorly from vulva (Fig. 1D). Ovaries extending slightly anterior to anus level (Fig. 1H). Eggs in uterus oval, thick-walled (4–5 wide), smooth; larvated eggs 35–42 × 23–27 (34–41 × 25–27) (Fig. 1D). Tail conical, 73–102 (73) long, with lateral phasmids and knob-like appendage at tip, 7–9 long, separated from body by a narrow constriction (Figs 1H, 3G, H).

##### Etymology.

The specific name of this nematode relates to the collection locality (Palmyra Atoll).

##### Type-host.

*Albula glossodonta* (Forsskål) (Albulidae).

##### Site of infection.

Intestine.

##### Type-locality.

Palmyra Atoll, Eastern Indo-Pacific Ocean.

##### Prevalence and mean intensity.

54.2 and 3.2 ± 4.0 (*n* = 24).

##### Specimens deposited.

Holotype and paratype (in SEM stub) specimens in the Helminthological Collection of the Institute of Parasitology, Academy of Sciences of the Czech Republic, Ceske Budejovice (no. N-1073) and CHCM no. 633 (allotype) (1 vial, 1 specimen ♀).

##### Remarks.

According to Moravec (2007), Moravec and Justine (2009), and Moravec and Klimpel (2009), the genus *Spinitectus* is one of the 24 valid genera within the family Cystidicolidae. This genus is represented by a large number of species described mainly from freshwater and marine fishes (Moravec et al. 2002) and includes the monotypic subgenus Paraspinitectus (Moravec and Justine 2009).

Due to the presence of markedly reduced pseudolabia in the oral opening and a body covered by spinose rings, the nematodes herein described were assigned to the subgenus Paraspinitectus, as diagnosed by Moravec and Justine (2009). The subgenus was created based on the structure of the oral opening of a lone female nematode reported as Spinitectus (Paraspinitectus) sp. collected from *A. glossodonta*, off New Caledonia. *Spinitectus beaveri*, a species originally described from *Albula vulpes* (Linnaeus) (Albulidae) in Biscayne Bay, Florida (Overstreet 1970) and later examined by SEM by Jilek and Crites (1982), had similar structure to the oral opening, and thus became the type species of the subgenus. The former was reported in the same host and geographical region (southern Pacific Ocean), while the latter was described from a congeneric host (*A. vulpes*) and different geographical region (off Florida). They both have very similar morphological characteristics to S. (P.) palmyraensis sp. nov., although, the new species differs from S. (P.) beaveri in the length of right spicule (673–766 vs 390–430 mm), vestibule (188–213 vs 80–90 mm), different pattern in the distribution and number of spinose rings, position of nerve ring (between rings 9–10 vs 3–7), and number of caudal papillae. The new species differs from Spinitectus (P.) sp. in the position of nerve ring (between rings 10–11), excretory pore (rings 9–10 vs 14–15) as well as the distribution pattern of spinose rings.

Interestingly even though the three species within *Paraspinitectus* occur in closely related hosts, morphological differences are evident among species of *Spinitectus* parasitizing albulid fishes around the world. Potentially, ecological differences of their hosts or habitats are substantial enough to select for this interspecific variability.

Morphological features as reduced pseudolabia, small triangular sublabia in the oral opening, trifurcated deirids (probably also present in Spinitectus (P.) sp.), and spinose rings with two different patterns of arrangement are common to the three known forms assigned to the subgenus. These characteristics support the validity of the subgenus Paraspinitectus and highlight the need for detailed SEM examination of cystidicolid specimens to determine the substantial intraspecific differences when comparing among species.

Within the Cystidicolidae there is a recently created genus, *Ascarophisnema* Moravec & Justine, 2010 with remarkable similarities with the new species. Similarities between the *Ascarophisnema* and *Spinitectus* include trifurcated deirids (only reported in these two genera), the structure of the oral opening, and the number and distribution of caudal papillae. Although the presence of spinose rings in *Spinitectus* clearly set it apart from *Ascarophisnema*. Probably, both genera are closely related but a phylogenetic analysis using molecular and morphological data is needed to clarify this situation.

This is the second nominal species reported within the subgenus Paraspinitectus and represents a new geographical record (southern Pacific region), since the previous report was a generically identified female from the same host species.

### Family Philometridae Baylis & Daubney, 1926

#### Philometra pellucida

Taxon classificationAnimaliaSpiruridaCystidicolidae

(Jägerskiöld, 1893) Yorke & Maplestone, 1926

B59D79BD-1A3E-5692-8BC0-79D1A2188959

##### Description.

Gravid female (3 specimens, measurements of 3 subgravid females in parentheses): large, yellowish nematodes, 111.42–140.70 (37.84–48.64) mm long, 0.94–3.29 (0.336–0.649) mm wide, with smooth cuticle and both ends rounded. Esophagus with anterior bulbous inflation, 220–267 (147–181) long and 163–182 (130–162) wide, with spacious lumen. Esophagus 1.59–2.06 (1.41–1.50) mm long, with dorsal esophageal gland not well demarcated, extended anteriorly to level of nerve ring. Esophageal gland with cell nucleus located at mid-length, at 0.915–1.07 (–) mm from anterior extremity. Small ventriculus 80–111 (50–80) long and 79–142 (75–85) wide, opening into intestine through valve. Nerve ring encircling esophagus just posterior to its anterior bulb, 279–407 (239–246) from anterior body end. Deirids and excretory pore not visible. Intestine brownish, straight and almost reaching the posterior end of body, forming ligament 325–690 (547–594) long, attached to the body wall near the caudal end. Ovaries extending near both ends of body. Uterus filled with larval mass (in gravid females) and developing embryos and eggs (in subgravid females). Larvae from uterus 345–492 long, with elongated tail. Posterior end of body rounded.

##### Host.

*Arothron hispidus*.

##### Site of infection.

Body cavity.

##### Prevalence and mean intensity.

40 and 20.8 ± 31.4 (*n* = 15).

##### Specimens deposited.

CHCM no. 634 (voucher) (1 vial, 1 specimen ♀).

##### Remarks.

Due to the presence of the anterior inflation of the esophagus, weakly demarcated esophageal gland, its occurrence in the body cavity of a congeneric fish host (*A. hispidus*) and close geographical region (Southeastern Pacific Ocean), these females were identified as *P. pellucida*. Gravid females showed similar morphometric features than those reported by Jägerskiöld (1893, 1894). They represent new host (*A. hispidus*) and geographical records (Palmyra Atoll).

## Discussion

The present study is the first detailed survey of the diversity and ecological attributes of the parasitic nematodes infecting fishes at Palmyra Atoll. Consistent with observations of the monogenean and parasitic copepod fauna of Palmyra Atoll fishes (Vidal-Martinez et al. 2017; Soler-Jiménez et al. 2019), parasitic nematode species richness at Palmyra Atoll appears low (10 species in 44 host fish) compared with others Indo-Pacific regions. In fact, several of the fish species we examined (16 of 44) were not parasitized by nematodes at all, even with large sample sizes for some fish species (e.g. *Osteomugil engeli* (Bleeker) (Mugilidae) *n* = 63, *Istigobius ornatus* (Rüppell) (Gobiidae) *n* = 26). Other fish species such as *C. melanopterus*, *C. melampygus*, *C. papuensis*, and *E. vaigiensis* with a single species of nematodes infecting them in this study (Table 1), have previous records of *Terranova* type II (larvae), *Anisakis typica*, *Hysterothylacium* type II (larvae), and *Camallanus carangis* (Deardorff et al. 1982; Moravec et al. 2006; Shamsi et al. 2011; Jabbar et al. 2012). Likewise, the nematode *Spirocamallanus colei* has been reported from *A. triostegus* (Rigby et al. 1997).

At a broad geographical scale, the most likely hypothesis to account for the paucity of parasitic nematodes at Palmyra Atoll is its geographical remoteness. Indeed, Palmyra Atoll apparently would show a pattern similar to that suggested by the island biogreography theory, where the large distance from the presumed centre of origin of Indo-West Pacific fishes and their parasites (the Austro-Malayan-Philippine region) would result in a low number of both fish and nematode parasite species. Further support for this explanation is the low species richness of fishes of the Line Islands, including Palmyra Atoll (Gosline 1971) compared to other coral atolls in the Indo-West Pacific region (e.g. Adler 1992). A similar pattern has been suggested for other groups of parasites of marine fish from the lagoonal flats of Palmyra Atoll such as monogeneans and parasitic copepods (Vidal-Martínez et al. 2017; Soler-Jiménez et al. 2019).

In our samples from the lagoonal flats of Palmyra Atoll, of the 10 nematode species recovered, seven were in the adult stage and three were larvae (Table 2). However, from the 43 fish species sampled, 24 were infected by larval stages of *Pulchrascaris* sp., followed by *Hysterothylacium* infecting seven fish species, *Pseudascarophis* sp. in three fish species, and *Cucullanus* in one species. The rest of the nematodes in Table 2 were adults and infected only one host species in low numbers. Because this is the most important pattern in the present study, it frames the rest of our discussion in the context of the life cycles of these nematodes.

The most likely explanation for the numerical dominance of the larval stages of nematodes is the lack of fishery activity at Palmyra Atoll and the substantial biomass of large, piscivorous sharks and ray-finned fishes (Lafferty et al. 2008). That means that the life cycles and transmission of nematodes of both elasmobranch and bony fishes acting as definitive hosts occur given the lack of selective removal of these hosts. This pattern at Palmyra Atoll agrees with the findings of Lafferty et al. (2008) for Kiritimati Islanad and Wood et al. (2015) at the Line Islands in the equatorial Pacific, as well as Marzoug et al. (2012) in the Mediterranean Sea, and Vidal-Martínez et al. (2019) in the Yucatan Peninsula, Gulf of Mexico. These authors have suggested that the completion of the life cycles of helminths such as cestodes using sharks as definitive hosts could be at risk due to overfishing. In the present study we have an opposite pattern where the lack of removal of the definitive hosts is the most likely explanation for the high number of larval nematodes using fishes as second intermediate hosts. We observed a similar pattern of abundant larval metacercarial stages in fishes at Palmyra (Vidal-Martínez et al. 2012).

The life cycle of the members of the *Pulchrascaris* genus is unknown but being an anisakid nematode, it should include small marine crustaceans as first intermediate hosts, fishes as second intermediate hosts and elasmobranch fishes as definitive hosts (Deardorff 1987; Moravec et al. 1995). In our study, the black tip shark *C. melanopterus* was infected with *Pulchrascaris chiloscylli*, and this shark is clearly acting as definitive host. All other 24 bony fish species in Table 2 are most likely acting as second intermediate host of *P. chiloscylli* and all other potential species of the same genus that could be present at Palmyra Atoll.

*Hysterothylacium* sp. larval stages were parasitizing seven fish species from the intertidal lagoon at Palmyra Atoll (Table 2). This nematode species is also a member of the Anisakidae (Moravec et al. 1995; Vidal-Martínez et al. 2001). Therefore, its life cycle should include small marine crustaceans as first intermediate hosts, marine bony fishes as second intermediate hosts, and carnivorous marine fishes as definitive hosts (Moravec et al. 1995; Vidal-Martínez et al. 2001). It is not surprising to find a long list of infected hosts with larvae of *Hysterothylacium* sp. in Palmyra Atoll because there are at least 60 marine fish species acting as intermediate hosts from nearby locations such as the Hawaiian Islands (Palm and Bray 2014).

*Pseudascarophis* sp. was also an adult nematode infecting three fish species (Table 2). Unfortunately, most of the material was lost, and the present description was based in only one gravid female. The life cycles of this species and that of the adult nematode *S. palmyrensis* sp. nov. are unknown. However, both nematodes belong to the family Cystidicolidae, from which several life cycles have been described. Briefly, eggs of Cystidicolidae contain fully developed first stage larvae, and crustaceans such as shrimps, crabs, and amphipods, as well as nymphal stages of aquatic insects (probably Ephemeroptera), act as first intermediate hosts. In these intermediate hosts, the nematodes have two molts, and fish acquire third stage larvae when they eat infected crustaceans (Anderson 2000).

The life cycle of the unidentified nematodes of the family Capillaridae is also unknown. However, based on the extant literature on the life cycles of the members of this family, it is likely that they use oligochaetes as first intermediate hosts (Kutzer and Otte 1966). Several authors, experimentally fed fish (*Salmo gairdneri*) with infected oligochaetes and obtained mature capillarid specimens of *Schulmanela petruschewskii* after six months (Kutzer and Otte 1966; Anderson 2000; Moravec 2001).

There were three species of the genus *Cucullanus* infecting fishes from the sand flats of Palmyra Atoll (Table 2), two as adults and a one a larva. Knowledge on the life cycle of the members of this genus is scarce (Anderson 2000). However, there is evidence to suggest the use of an intermediate host has been replaced by a histotropic phase in the definitive host (Gibson 1972; Anderson 2000). This means that if the fish eats eggs or first stage larvae occuring in the environment, the rest of the development occurs entirely in the definitive host.

The life cycle of *Philometra pellucida* (Table 2) is unknown, but information on other members of this genus suggest that copepods should be the first intermediate hosts (Moravec 1998; Anderson 2000). Once the fish eats infected copepods, the parasite develops until reaching sexual maturity in their preferred microhabitat (in this case, in the body cavity).

In conclusion, despite the relatively low species richness of parasitic nematodes in the lagoonal flats at Palmyra Atoll, which is due to its remotedness, there were very interesting patterns at the local level, especially those related with the numerical dominance of larval stages of nematodes. Apparently, the lack of fishing at the atoll since 2001 (https://www.fws.gov/refuge/palmyra_atoll/) and its selective removal of definitive hosts such as sharks and piscivorous bony fishes applied is the most likely explanation for the high number of larval nematodes using fishes as second intermediate hosts.

## Supplementary Material

XML Treatment for Pulchrascaris chiloscyllii

XML Treatment for Pulchrascaris

XML Treatment for Hysterothylacium

XML Treatment for Capillariidae

XML Treatment for Cucullanus bourdini

XML Treatment for Cucullanus oceaniensis

XML Treatment for Cucullanus

XML Treatment for Pseudascarophis

XML Treatment for Spinitectus (Paraspinitectus) palmyraensis

XML Treatment for Philometra pellucida
